# Innovative Approaches to Fucoxanthin Delivery: Characterization and Bioavailability of Solid Lipid Nanoparticles with Eco-Friendly Ingredients and Enteric Coating

**DOI:** 10.3390/ijms252312825

**Published:** 2024-11-28

**Authors:** Lijun Ding, Xiao Luo, Qingyue Xian, Sishi Zhu, Weijia Wen

**Affiliations:** 1 Thrust of Bioscience and Biomedical Engineering, The Hong Kong University of Science and Technology (Guangzhou), Guangzhou 511400, China; lding472@connect.hkust-gz.edu.cn; 2 Department of Physics, The Hong Kong University of Science and Technology, Kowloon 999077, Hong Kong, China; xluoay@connect.ust.hk; 3 Thrust of Advanced Materials, The Hong Kong University of Science and Technology (Guangzhou), Guangzhou 511400, China; qxianaa@connect.hkust-gz.edu.cn (Q.X.); szhu260@connect.hkust-gz.edu.cn (S.Z.)

**Keywords:** fucoxanthin, solid lipid nanoparticle (SLN), bioavailability, encapsulation, enteric coating, nano drug delivery

## Abstract

Fucoxanthin (FN), a carotenoid derived from brown seaweed and algae, offers significant health benefits. However, its unique structure leads to challenges in stability and bioavailability. To overcome these issues, we successfully encapsulated fucoxanthin in solid lipid nanoparticles (SLNs) utilizing health-safe materials, achieving remarkable results. SLNs exhibited a nanoscale size of 248.98 ± 4.0 nm, along with an impressive encapsulation efficiency of 98.30% ± 0.26% and a loading capacity of 5.48% ± 0.82% in lipid. The polydispersity index (PDI) was measured at 0.161 ± 0.03, indicating a narrow size distribution, while the high negative zeta potential of −32.93 ± 1.2 mV suggests excellent stability. Pharmacokinetic studies conducted in Sprague–Dawley rats revealed an exceptional oral bioavailability of 2723.16% compared to fucoxanthin crystals, likely attributed to the enhanced stability and improved cellular uptake of the SLNs. To further improve bioavailability, we creatively applied enteric coatings to the freeze-dried SLNs, effectively protecting fucoxanthin from gastric degradation, which is supported by in vitro digestion results. These findings underscore the potential of SLNs as a superior delivery system for fucoxanthin, significantly enhancing its therapeutic efficacy and broadening its application in the food and pharmaceutical industries.

## 1. Introduction

Fucoxanthin was a characteristic carotenoid compound present in seaweeds such as Phaeophyta and microalgae [1]. The compound has been widely investigated due to its remarkable biological protective properties for human health. As a distinctive carotenoid, fucoxanthin showed many bioactivates: high antioxidant activity, anti-obesity, anti-diabetes, anti-inflammatory, anti-cancerous, and hepatoprotective properties as well as cardiovascular and cerebrovascular protective effects [2]. Therefore, fucoxanthin may have potential value in preventing and treating lifestyle-related diseases, such as obesity, diabetes, cancer, cardiovascular disease, and other chronic diseases. It is a promising medicinal and nutritional ingredient [3]. Further studies have demonstrated that fucoxanthin is a safe pharmaceutical ingredient, based on various toxicity studies [4,5]. Nevertheless, fucoxanthin (FN) encounters obstacles pertaining to stability and bioavailability because of its distinctive structure, most notably the allene bond. Factors such as light, heat, and oxygen can contribute to its degradation and hinder its absorption and bioavailability in the body [6,7]. Sugawara et al. investigated the bioavailability of fucoxanthin together with other carotenoids and demonstrated that fucoxanthin has one of the lowest (~50 pmol/mg protein) bioavailability among other studied carotenoids [8]. Furthermore, fucoxanthin is not readily absorbed because it is hydrophobic in nature. Gastric juices of the stomach would degrade fucoxanthin before reaching the intestine. To overcome these problems, different encapsulation techniques have been previously explored. 

The materials for the delivery systems of fucoxanthin include single materials and composite materials, and the composite materials mainly include protein–polysaccharide, protein–protein, polysaccharide–polysaccharide, protein–lipid, and polysaccharide–lipid [9,10,11]. Colloidal structures for fucoxanthin include nanoparticles, microcapsules, emulsions, gels, coacervates, and nanofibers [12]. Emulsion systems, spanning a range of sizes from micro to nano, have demonstrated a reduction in the degradation of FN when utilizing carriers such as triglycerides and canola oil, when exposed to heat, oxygen, and light [13]. However, their elevated surface-to-volume ratio can lead to increased degradation during storage due to contact with the aqueous phase. The use of lipid-based nanotechnology, encompassing nanoliposomes, nanosuspensions, nanoemulsions, and solid lipid nanoparticles, has the potential to address the degradation and absorption challenges associated with fucoxanthin [14,15,16,17,18]. Among these, SLNs are a popular model due to the advantages they offer over other encapsulation systems. SLN is a colloidal system made up of lipids such as steroids, fatty acids, partial glycerides, waxes, and triglycerides, which remain solid at room temperature [19]. In comparison to liquid lipids, SLNs have demonstrated higher chemical stability compared to liquid lipids, attributed to reduced diffusion between the lipid core and the aqueous phase [20,21,22]. For example, Amira et al. enhanced oral bioavailability of simvastatin using polymer-coated solid lipid nanoparticles [23]. Nevertheless, the utilization of SLN as an encapsulation method for fucoxanthin remains scarce. Quan et al. [24] developed characterized fucoxanthin-loaded microspheres and Yang et al. [25] designed complex carriers composed of gum Arabic/gelatin microcapsules and alginate hydrogel beads for oral delivery of fucoxanthin. However, the particle size showed in the micrometer range with a limited in vivo study. Hindupur et al. [26] fabricated chitosan–glycolipid hybrid nanogels containing fucoxanthin improved the bioavailability. In addition, another study demonstrated that bioavailability of fucoxanthin increased twofold when it was coated with casein and chitosan [27]. Chen et al. [28] developed Fucoxanthin-loaded palm stearin and cholesterol-based solid lipid nanoparticle-microcapsules, with an average particle size of 1154 ± 54 nm, which falls in the large nano scale range. Consequently, there is scope for enhancing the particle size and bioavailability of fucoxanthin.

The objective of our research is to utilize green ingredients and employ the SLN method with mixed lipid matrices to produce small nanoscales and freeze-dried FN-loaded SLNs. Natural medium-chain triglycerides (MCTs), mainly sourced from coconut oil and palm kernel oil, have melting points that are at or just below body temperature, suggesting good biocompatibility and low toxicity while facilitating the release of bioactive compounds during digestion. Coconut oil with cis unsaturated fats would alter blood lipid profiles in a manner consistent with a reduction in risk factors for cardiovascular disease [29]. Consequently, coconut oil and palm oil were selected as the lipid sources for this study. Exploring solid lipids from natural sources, glyceryl monostearate (GMS) composed mainly of stearic acid was added to provide chemically stable SLNs with controlled retention of lipophilic bioactive during storage [30,31,32]. Phospholipids were selected to add into the molten lipid phase to the stabilization of the lipid matrix and use a Tween 80 as surfactant to reduce interfacial tension. This combination creates a mechanical and/or electrical barrier that prevents particle coalescence during SLN formation and aggregation during storage.

This approach is expected to significantly improve bioavailability and enhance product stability for storage. Furthermore, the FN–SLNs were effectively protected from degradation by gastric fluid, marking the first use of an enteric coating. This optimal encapsulation method for FN offers promising applications in functional foods and pharmaceuticals while providing valuable insights into nano-delivery systems for hydrophobic compounds.

## 2. Results

### 2.1. Selection of Lipids, Surfactants, and Homogenization Cycles

Finding the right balance is crucial for achieving the desired particle size and encapsulation efficiency (EE%). For preliminary studies, lipid components, emulsifier type and concentration, and the number of homogenization cycles were selected through an orthogonal experiment (Table 1). Solubility tests indicated that FN was soluble in both hydrogenated palm oil (HPO) and coconut oil at concentrations ranging from 1% to 10%, with 1% concentration chosen for further experiments.

The analysis of the orthogonal test results (Table 2) revealed that the particle size of FN was smaller when coconut oil was used as the lipid source in combination with Tween 80 as the emulsifier. Given that coconut oil is body-friendly and produces relatively small particle sizes, it was selected for subsequent experiments. The optimal surfactant concentration was determined to be 1–1.5% Tween 80, with the ideal number of high-pressure homogenization (HPH) cycles set at 3 to 4. Results showed that FN–SLNs achieved a nano-range particle size with a high percentage of coconut oil, minimal surfactant concentration, and sufficient homogenization cycles. While increasing the number of HPH cycles reduced particle size, no further decrease was observed beyond four cycles. The polydispersity index (PDI) of all samples was low, ranging from 0.178 ± 0.012 to 0.302 ± 0.020, and the zeta potential values for all nine samples exhibited a high negative magnitude.

### 2.2. Formulation of FN–SLN

Based on the preliminary results in Section 2.1, fucoxanthin (10% in lipid) was successfully encapsulated using a formulation of 95% coconut oil and 5% glycerol monostearate (GMS), followed by the addition of 1.25% Tween 80 and 1.25% soy lecithin, with a HPH cycle of 4. This formulation yielded a homogenized, orange-colored FN–SLN dispersion.

### 2.3. Characterization of FN-SLN

#### 2.3.1. Morphology Analysis

**Figure 1:**
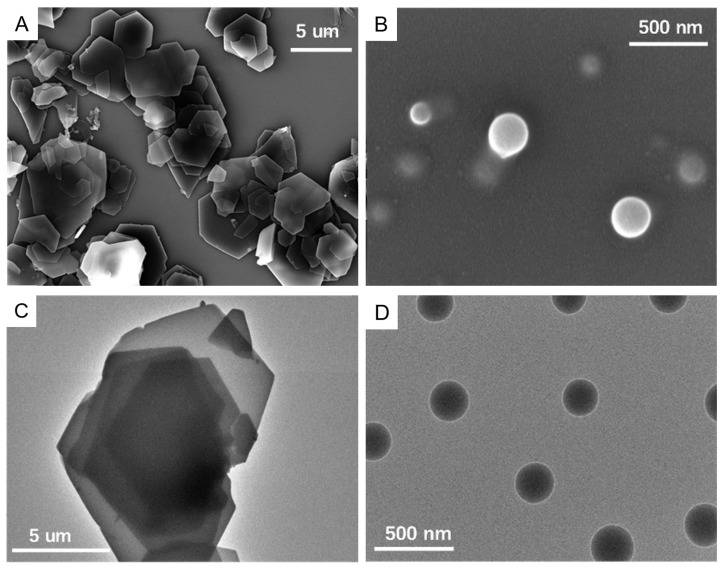
TEM of FN crystal (**A**) and FN–SLN (**B**); SEM of FN crystal (**C**) and FN–SLN (**D**) at different magnifications.

The morphology of both FN and FN–SLNs was observed via both scanning electron microscopy (SEM) and transmission electron microscopy (TEM). Figure 1A,C illustrates that, due to the low water solubility of FN, it existed in its crystalline form with a distinct inherent polyhedron shape in the aqueous medium, exhibiting an overall average length exceeding 5 µm. However, upon formulating FN into FN–SLN, the resulting nanoparticles exhibited a significantly reduced size in the nanometer range and a distinct round shape, Figure 1B,D. At 179 K magnification, the surface was observed to be relatively smooth. As shown in these micrographs, FN–SLNs exhibited different morphologies over the FN crystals, which have the potential to increase surface area and improve solubility and enhance drug delivery. Therefore, FN–SLN showed considerable potential for improved therapeutic efficacy and bioavailability.

#### 2.3.2. Particle Size, PDI, and Zeta-Potential

**Figure 2:**
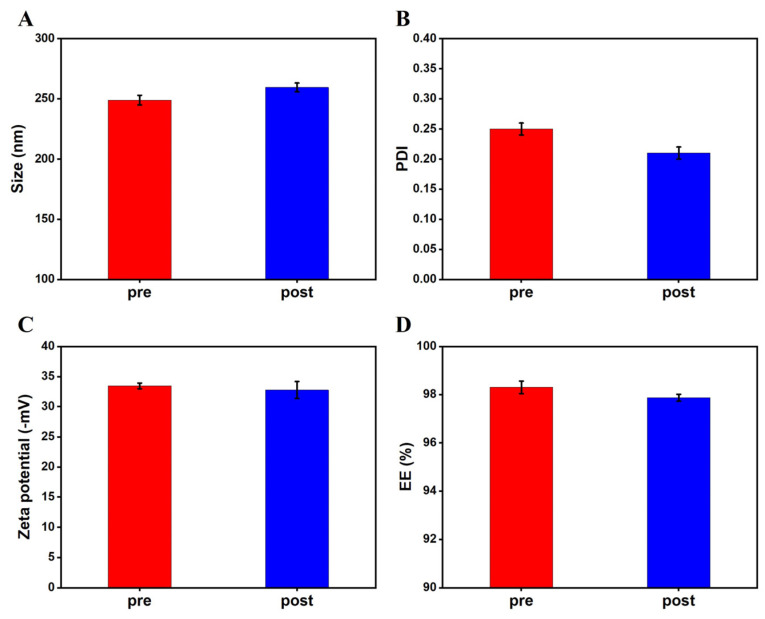
Characterization of FN-SLN for pre-lyophilization and post-lyophilization (*n* = 3). (**A**) Comparison of particle size. (**B**) Comparison of PDI (Polydispersity Index). (**C**) Comparison of Zeta potential. (**D**) Comparison of EE% (Encapsulation Efficiency).

The mean particle diameter of the FN–SLN was observed within the lower nanoscale range, measuring 248.98 ± 4.0 nm (Figure 2 pre). This particle size suggests effective formulation of the FN–SLN, which is crucial for their intended applications in drug delivery and therapeutic formulations. The PDI values of FN–SLN were found to be 0.253 ± 0.07. The zeta potential of FN–SLN revealed to be negatively charged, with a value of −33.45 ± 0.48 mV. The recorded zeta potential value exceeded ±30 mV, which is the threshold required for a stable SLN system [33,34]. Therefore, in terms of colloidal stability, FN–SLN is demonstrated to be homogeneous and stable. The outcome of EE and LC are 98.30% ± 0.26% and 1.05% ± 0.16%, respectively, indicating that a significant portion of FN was effectively encapsulated. The results demonstrate that the encapsulation process maintains the desired characteristics for optimal performance. 

### 2.4. Lyophilizated FN–SLN Evaluation

#### 2.4.1. Lyophilization

To facilitate freeze-drying and achieve a free-flowing powder without significant increases in particle size, three cryoprotectants were evaluated at different concentrations, as summarized in Table 3. When the concentration of the cryoprotectant was increased to 20%, formulations using glucose and mannitol resulted in hygroscopic and lumpy powders after freeze-drying. In contrast, the SLNs freeze-dried with trehalose yielded a dry and free-flowing powder. Based on these findings, 20% trehalose was selected as the optimal cryoprotectant to achieve the desired powder characteristics.

#### 2.4.2. Reconstitution

In this study, the particle size, polydispersity index (PDI), zeta potential, and encapsulation efficiency (EE%) were evaluated during the reconstitution of FN–SLN freeze-dried powders in contrast to non-freeze-dried SLNs, as detailed in supplementary (Table S1). After lyophilization, as shown in Figure 2, the nanoparticle characteristics presented a relatively small change in size (4.23%), Zeta potential (2.06%), and EE% (0.44%), along with a notable decrease in polydispersity index (PDI) (about 16.67%). The reduction in PDI might be attributed to the rearrangement and optimized interactions of lipid and emulsifiers, as well as the elimination of water-related interference, leading to a more uniform nanoparticle size distribution. These findings highlight a novel aspect of our research, contributing to the understanding of SLN performance following freeze-drying.

#### 2.4.3. Dispersion

**Figure 3:**
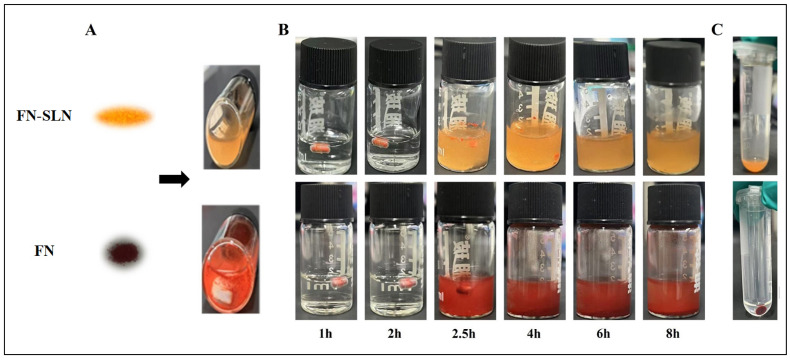
(**A**) Dispersion of Fucoxanthin-Solid Lipid Nanoparticles (FN–SLN) and Fucoxanthin in ultra-pure water. (**B**) Dissolution behavior of Fucoxanthin-Solid Lipid Nanoparticles (FN–SLN) and Fucoxanthin with enteric-coated capsule in Simulated Gastric Fluid (SGF) and Simulated Intestinal Fluid (SIF). (**C**) Supernatant appearance after standing for 2 h.

After freeze-drying, FN–SLN powders with a yellow to orange color were obtained, as shown in Figure 3A. This figure also visually illustrates the distinctive characteristics of FN in its two forms. The dispersion of FN was found to be inadequate, leading to the formation of aggregates on the walls and bottom of the tube, indicating a lack of stability in the solutions. In contrast, the FN–SLN formulation demonstrated stable dispersion in water, reflecting effective solubilization and uniform distribution of the nanoparticles. This uniformity in nanoscale size indicates that the formulation and processing techniques employed are effective in producing FN–SLNs with consistent particle sizes, which is crucial for their intended applications in drug delivery and therapeutic formulations.

#### 2.4.4. Particle Size Stability After Storage

Table 4 presents data on the stability of freeze-dried FN–SLNs stored at 4 °C for 90 days, evaluating particle size, PDI, zeta potential, and encapsulation efficiency (EE%). The particle size increased slightly from 259.51 ± 3.65 nm on Day 1 to 303.53 ± 5.18 nm by Day 90 with an average daily increment of about 0.49 nm, which indicates that FN–SLNs maintained stable dimensions throughout the study period, and this minimal change highlights the effectiveness of the formulation, reinforcing the suitability for long-term applications. The PDI values remained consistently low, showing only slight fluctuations and reflecting a uniform size distribution of the nanoparticles despite the size increase. Zeta potential values ranged from −32.76 mV to −39.69 mV and had absolute values constantly above 30 (despite some oscillations), demonstrating high stability due to strong electrostatic repulsion that effectively prevented aggregation. Furthermore, the EE% remained high throughout the study, ranging from 95.82% to 97.87% with a small decrease of around 1.59% over 90 days, suggesting good retention of fucoxanthin within the nanoparticles and negligible practical implications due to this change. Overall, these results underscore the strong stability and encapsulation efficiency of FN–SLNs over time and suggest that the nanoparticles maintain a relatively stable state during storage.

#### 2.4.5. In Vitro Release Evaluation

**Figure 4:**
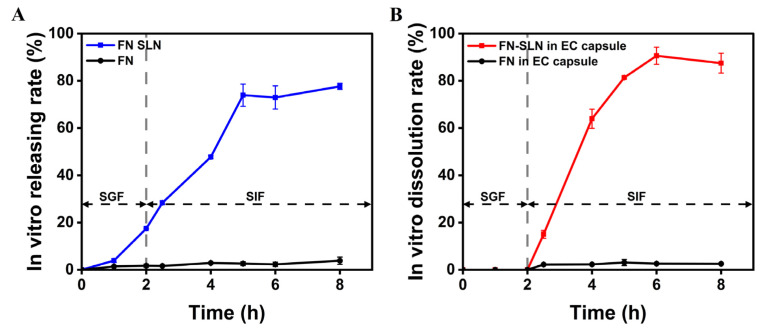
In vitro release study: (**A**) In vitro release curve of the formulated FN–SLN and FN crystal in simulated gastrointestinal fluid; (**B**) In vitro dissolution curve of FN–SLN in an enteric-coated capsule vs. FN crystal in an enteric-coated capsule in simulated gastrointestinal fluid (*n* = 3).

This graph (Figure 4A) depicts the in vitro release profiles of fucoxanthin with our formulated FN–SLN compared with FN. The release rates are shown under simulated fluid conditions, which mimic the gastrointestinal environment. Under simulated gastric fluid (SGF) conditions (pH around 1.2–2.0), the FN–SLN formulation exhibited a rapid initial burst release, with nearly 17.44% of the FN released within the first 2 h. By changing into the simulated intestinal fluid (SIF) environment (pH around 6.8–7.5), the release profiles diverged further. The FN–SLN formulation maintained a sustained release pattern, with 77.65% of the FN released over the 8-h observation period. This suggests that the FN would be readily available for absorption in the upper gastrointestinal tract. This gradual release in the intestinal phase could enhance the bioavailability of FN by providing a more consistent and efficient absorption. In contrast, the FN crystal formulation exhibited significantly lower release rates in both SGF and SIF conditions, which reached their highest at 2.88%. This highlights the advantages of the nanoparticle-based delivery system in improving the dissolution and release kinetics of the lipophilic FN compound.

#### 2.4.6. In Vitro Dissolution Study with Enteric Coating

Figure 3B describes the in vitro drug-dissolution behavior of two different drug formulations in enteric-coated capsules under simulated gastric and intestinal conditions over an 8-h period. For the FN–SLN, after 2 h, the capsule has completely dissolved in the gastric fluid, allowing the drug to be released and resulting in an orange-red-colored solution. Over the subsequent 4–6 h, the drug remains fully dissolved and dispersed in the intestinal fluid, with the solution color remaining relatively consistent. This demonstrates that the enteric-coated capsule design can initially prevent drug release in the gastric environment and then gradually release the drug over the 6-h period. In contrast, for FN, the drug is similarly released from the dissolving capsule into the gastric/intestinal fluid. However, the FN does not readily dissolve or disperse well in the fluid. Instead, the drug tends to precipitate and settle at the bottom of the vial, rather than remaining evenly dispersed in the solution (Figure 3C).

Compared to the non-enteric-coated FN–SLN, the addition of an enteric-coated capsule to the FN–SLN formulation demonstrated differences in release behavior during the initial 2 h in SGF. As shown in Figure 3B, the FN–SLN formulation encapsulated within the enteric-coated capsule exhibited no release during the initial 2-h period in gastrointestinal conditions. This delayed release in the gastric environment is a desirable characteristic, as it effectively protects the sensitive FN compound from the acidic conditions of the stomach, thereby safeguarding it from potential gastric fluid-induced degradation. Upon switching to SIF, the FN–SLNs exhibited a rapid release profile, achieving 90.60% dissolution within the observed 6-h period, an improvement from 77.65% (Figure 3A). However, despite the limited release in the initial 2-h SGF phase, the unformulated FN crystals maintained a consistently low release rate throughout the 8-h observation period, even under SIF conditions. The limited solubility and tendency for precipitation of the FN crystals hinder their effective release and absorption in the gastrointestinal tract.

Applying an enteric-coated capsule for our sensitive FN compound can be a valuable strategy to improve the therapeutic potential of fucoxanthin with its enhanced release characteristics.

#### 2.4.7. In Vitro Cytotoxicity Assessment

**Figure 5:**
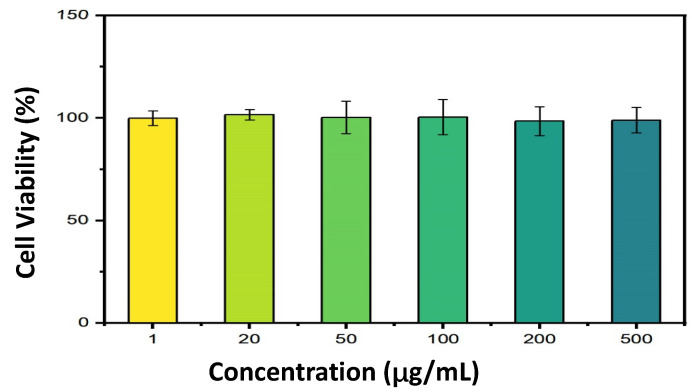
Cell viability (%) at different concentrations (μg/mL) of FN-SLNs (*n* = 6).

Despite the formulation being constituted of eco-friendly ingredients, the assessment of its cytotoxicity remains indispensable. In this study, the cytotoxicity of FN–SLNs on HaCaT cells was determined by the CCK-8 test. Data was presented in Figure 5. The cells interacted with 1 μg/mL, 20 μg/mL, 50 μg/mL, 100 μg/mL, 200 μg/mL, and 500 μg/mL of the relevant FN–SLN formulation in the experiment. As can be seen, the cell-viability percentages for HaCaT cells are approximately maintained around 100% (Supplementary Table S2); no cytotoxicity effect was observed on HaCaT cells.

#### 2.4.8. In Vivo Pharmacokinetics Evaluation

**Figure 6:**
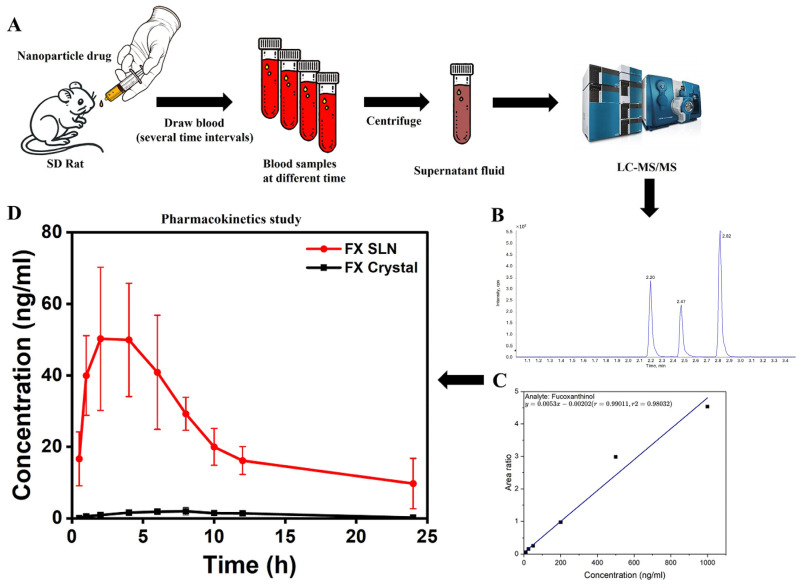
(**A**) In vivo animal study process flowchart (**B**) Chromatogram of three analytes in sequence from left to right: Fucoxanthinol, Hydroxyprogesterone caproate and Fucoxanthin. (**C**) Calibration curve used for FNOH quantitation in rat plasma (**D**) Plasma concentration-time profiles of FNOH in rats after oral dosing of FN and FN–SLN (10mg/Kg/FN) (*n* = 6).

The in vivo pharmacokinetics results demonstrated that fucoxanthin was predominantly transformed into fucoxanthinol (FNOH) in the rat intestine, involving lipase and cholinesterase, as the concentration of FN in rat plasma was below the lowest limit of quantitation (LOQ) in our analysis. This process facilitated the hydrolysis of FN to a more absorbable metabolite, FNOH, which plays a pivotal pharmacological role [35]. A comparison of the pharmacokinetic data (Figure 6) for oral administration of FN crystals and FN–SLN (10 mg/kg) to rats reveals notable differences in their performance. Table 5 showed that the maximum concentration (C_max_) of FNOH was significantly higher in the FN–SLN group (53.63 ± 19.11 ng/mL) compared to the FN–Crystal group (2.14 ± 1.04 ng/mL), indicating enhanced absorption. Furthermore, the maximum FNOH concentration in rat plasma was reached more rapidly for A, with a T_max_ of 3.0 ± 1.15 h, indicating a more rapid absorption and utilization in comparison to B, for which the T_max_ was 7.0 ± 1.09 h. The area under the curve from zero to the last measurable concentration (AUC_0-last_) and from zero to infinity (AUC_0-inf_) were markedly greater for FN–SLNs (536.19 ± 164.77 ng·h/mL and 595.37 ± 207.17 ng·h/mL, respectively) compared to FN (19.69 ± 12.51 ng·h/mL and 43.99 ± 12.53 ng·h/mL), indicating a more extensive overall exposure to the active compound. The elimination half-life (T_1/2_) of FNOH was found to be shorter for FN–SLNs (5.29 ± 2.90 h) than for FN (14.46 ± 3.49 h). Furthermore, the clearance rate (CL) was markedly higher in the FN group (239.18 ± 63.28 L/h/kg) than in the FN–SLNs group (18.18 ± 6.08 L/h/kg). These findings indicate that the FN–SLN formulation markedly enhances the pharmacokinetic profile of FNOH, thereby increasing its bioavailability. The relative bioavailability of FN–SLN was found to be 2723.16% compared to fucoxanthin crystals, indicating a significant enhancement in absorption. This considerable increase in absorption implies enhanced therapeutic efficacy when FN–SLN is administered using our formulation. A high relative bioavailability is of great importance for the optimization of dosing regimens, the maximization of the drug’s efficacy, and the minimization of potential side effects.

## 3. Discussion

Fucoxanthin has exhibited a diverse range of potentially valuable pharmacological activities. However, its practical application within the medical domain has been considerably hampered by challenges such as low bioavailability and poor solubility [36]. In response to these obstacles and with the objective of unlocking its complete therapeutic potential, this study was centered on formulating and characterizing fucoxanthin—loaded solid lipid nanoparticles.

The development of the FN–SLN formulation was a meticulously executed process. Through an orthogonal experiment, coconut oil was selected as the lipid due to its favorable biocompatibility and its proficiency in generating small particle sizes. Tween 80 was identified as the optimal emulsifier, with a concentration range of 1–1.5% being determined as most suitable. Moreover, four high-pressure homogenization cycles were established as the ideal processing conditions. These precisely defined parameters led to the creation of FN–SLN with nanoparticles of a desirable size. The low PDI indicated a narrow size distribution, while a high zeta potential—specifically,33.45 ± 0.48 mV (exceeding ±30 mV)—guaranteed excellent colloidal stability. Additionally, a remarkably high EE% of 98.30 ± 0.26% was achieved, along with a drug loading of 5.48%.

Characterization studies further substantiated the potential of FN–SLN. The mean particle diameter of 248.98 ± 4.0 nm fell within the desired nanoscale range. SEM and TEM analyses revealed a significant reduction in size and a transformation in shape from FN crystals to round FN–SLN nanoparticles. This alteration is likely to enhance the surface area, thereby potentially improving solubility and drug delivery, and consequently augmenting therapeutic efficacy and bioavailability. The nanoparticles, being solid lipid nanoparticles, exhibited a relatively regular spherical shape and demonstrated no significant aggregation even after freeze-drying.

The evaluation of lyophilized FN–SLN provided valuable insights. Trehalose at a concentration of 20% was identified as the optimal cryoprotectant, as it yielded a free-flowing powder post-freeze-drying. After lyophilization, only minor changes were observed in particle size (4.23%), zeta potential (2.06%), and EE% (0.44%). Notably, a significant reduction in PDI (about 16.67%) was detected, which was likely attributable to the rearrangement of lipids and emulsifiers and the elimination of water-related interference.

Regarding the particle-size stability after storage, a slight increase in particle size was noted over a 90-day period, with an average daily increment of approximately 0.49 nm. This was accompanied by consistently low PDI values and a high absolute zeta potential value, all of which indicated the long-term stability of FN–SLN. The relatively small decrease in EE% (around 1.59%) over the 90 days further supported its suitability for long-term applications.

The in vitro release study clearly demonstrated the advantages of FN–SLN. In simulated gastric fluid, it exhibited a rapid initial burst release, with nearly 17.44% of FN released within the first 2 h. This was followed by a sustained release in simulated intestinal fluid, with 77.65% of FN released over the 8-h observation period. In contrast, FN crystals displayed significantly lower release rates in both SGF and SIF. This emphasized the enhanced dissolution and release kinetics of the lipophilic FN compound facilitated by the nanoparticle-based delivery system, which likely contributes to improved bioavailability. Through in vitro testing, FN–SLN also demonstrated excellent dispersibility in gastrointestinal fluid.

The in vitro dissolution study with enteric coating was equally revealing. The enteric-coated capsule effectively shielded FN–SLN from the stomach’s acidic environment, with no release occurring during the initial 2-h SGF period. Upon exposure to SIF, a rapid release profile was observed, with 90.60% dissolution achieved within 6 h, representing an improvement over the non-enteric-coated FN–SLN. In contrast, FN crystals maintained a consistently low release rate. This underlined the crucial role of enteric coating in safeguarding FN and facilitating its controlled release and absorption in the intestine, thereby maximizing bioavailability. The nanoparticles, when used in combination with enteric capsules, conferred good stability and prevented aggregation.

The in vitro cytotoxicity test, conducted on HaCaT cells using the CCK-8 assay, indicated no cytotoxicity, even at high FN–SLN concentrations. This attested to the safety of the formulation for potential therapeutic applications.

Finally, the in vivo pharmacokinetics study furnished conclusive evidence of FN–SLN’s enhanced performance. When compared to FN crystals, FN–SLN significantly increased the C_max_ of fucoxanthinol, reduced the time to reach maximum concentration, and increased the area under the curve (AUC_0-last_ and AUC_0-inf_). The relative bioavailability of FN–SLN was an astounding 2723.16% (27.2%) higher than that of fucoxanthin crystals. In comparison, other studies have also made contributions in the exploration of fucoxanthin bioavailability enhancement as detected through FNOH in plasma, such as approximately 6.66 and 4.39 times [37], 2.9 times [38], and yet another attaining 7.12 times [28]. However, our study has surpassed these achievements by attaining a remarkable enhancement in bioavailability, which shows the superiority of our approach.

## 4. Materials and Methods

### 4.1. Materials and Instruments

#### 4.1.1. Chemicals and Reagents

Fucoxanthin and fucoxanthinol were purchased from Sigma Aldrich (Saint Louis, MO, USA) (purity ≥ 99%). Hydroxyprogesterone caproate (purity ≥ 98%), the IS, was purchased from the National Institutes for Food and Drug Control (Beijing, China). Coconut oil, Tween 80 was purchased from Macklin Biochemical Co., Ltd. (Shanghai, China). Glyceryl monostearate was purchased from Aladdin Bio-Chem technology Co., Ltd. (Shanghai, China). Simulated Gastric Fluid-Sterile and Simulated Intestinal Fluid-Sterile were purchased from Phygene Biological technology Co., Ltd. (Fuzhou, China). Cell counting Kit-8 (CCK-8) was purchased from GlpBio Technology Inc. (Montclair, CA, USA). HACAT cell culture medium was purchased from Pricella (Pricella Biotechnology, Wuhan, China). The EUDRACAP^®^ preclinic enteric capsule was a generous gift from Evonik (Piscataway, NJ, USA). HPLC-grade acetonitrile and methanol were obtained from Thermo Fisher (Darmstadt, Germany). HPLC-grade formic acid was obtained from Roe Scientific Inc. (Powell, OH, USA). Ultrapure water was produced by a Millipore Milli-Q system (Millipore Corp., Billerica, MA, USA). All other reagents or solvents were commercially available and reagent-grade. Blank rat plasma was collected from healthy male Sprague–Dawley rats weighing 300 ± 20 g (Zhuhai BesTest Bio-Tech Co., Ltd., Zhuhai, China).

#### 4.1.2. Chromatographic and Mass Spectrometric Conditions

- Liquid Chromatography Conditions for In Vitro Study

Analyte separations were conducted on a UPLC-1290 system equipped with a Diode Array Detector (DAD) from Agilent Corp. (Milford, MA, USA). The analysis utilized a C18 analytical column (4.6 mm × 150 mm, 5.0 µm) from Agilent Technologies, Inc. (Santa Clara, CA, USA), which was maintained at a temperature of 25 °C. The mobile phase consisted of acetonitrile (A) and aqueous 0.1% formic acid (B) in a ratio of 90:10 (*v*/*v*). The flow rate was set at 1 mL/min, and the injection volume for each sample was 5 μL.

- Liquid Chromatography Coupled with Tandem Mass Spectrometry Conditions

The qualification and quantification of FN and FNOH in plasma samples was conducted using Nexera LC-40 (Shimadzu UPLC, Kyoto, Japan) coupled with AB 6500+ Q-trap mass spectrometry (ABSCIEX, Framinham, MA, USA), which was equipped with electrospray ionization (ESI). The mobile phase was composed of H_2_O (A: 0.1% formic acid and acetonitrile (B, 0.1% formic acid; A:B = 10:90, *v*/*v*) at a flow rate of 0.35 mL/min, and the injection volume was 5 µL. The analysis utilized a Kinetex EVO C18 column (2.1 mm × 100 mm, 2.6 μm) Phenomenex, Torrance, CA, USA), which was maintained at a temperature of 25 °C. The detection of the compounds (Table 6) was performed in positive ion mode under the following conditions: curtain gas at 35.0 L/h, collision gas medium, ion spray voltage at 5000 V, temperature at 400 °C, and ion source gases 1 and 2 both at 55 L/h. The total chromatographic time was 7 min, with the retention times of FN, FNOH, and the internal standard (IS) being 2.82, 2.47, and 2.20 min, respectively (Figure 6B and Figure S1). The data was acquired using AB Analyst 1.6.1 software (ABSCIEX, USA). Plasma samples were analyzed and calibrated through the FNOH (with IS) calibration curve y = 0.0053x − 0.00202 (R^2^ = 0.98032) Figure 6C and Figure S2).

### 4.2. Methods for SLN Preparation

#### 4.2.1. Lipid Phase Formation and Enrichment with FN

Heloísa Helena de Abreu Martins’s hot homogenization method [39] was employed with some modifications. Briefly, lipids were weighed and melted at a maintained temperature with water bath at 65 °C. GMS was added afterwards for dissolution by magnetic stirring for 15 min. The lipid phase consisting of solid fats was enriched with fucoxanthin. While the lipids were maintained at 65 °C, crystalline fucoxanthin powder was added, and hereafter the mixture was stirred for 10 min in the absence of light until a complete dissolution of fucoxanthin crystals was observed.

#### 4.2.2. Formation of Solid Lipid Nanoparticles

The lipid phases (5% *w*/*w*) were mixed with non-ionic surfactants and Mili-Q water with a high-speed blender (Ultra Turrax T25 homogenizer, IKA^®^ Works, Inc. Wilmington, NC, USA) working at 7800 rpm for 5 min. Afterwards, coarse emulsions were subsequently passed through a microfluidizer (MP-110 Microfluidics, Newton, MA, USA) at 100 MPa for 3–5 cycles, in order to reduce the particle size down to the nanometric range. The lipid phase, surfactant, and aqueous phase were kept at 65 °C before and throughout the entire emulsification process, subsequently cooled down to room temperature then to 4 °C for 3 h to allow the recrystallization of the lipid phase.

### 4.3. Particle Physicochemical Characterization

The physicochemical characteristics of SLNs, including mean particle size, polydispersity index, and zeta-potential, were assessed immediately after SLN formation, after freeze-drying, and during storage at 4 °C.

#### 4.3.1. Particle Size

The particle size was measured by light-scattering technique using a NanoBrook Omni (Brookhaven Instruments, Holtsville, New York, NY, USA). For dynamic light-scattering measurements the sample was diluted 1:199 in ultrapure water and the particle size was reported as z-average (nm).

#### 4.3.2. Zeta-Potential

The Zeta-potential (mV) was measured by phase-analysis light scattering (PALS) with a NanoBrook Omni laser diffractometer (Brookhaven Instruments, Holtsville, New York, NY, USA). Samples were diluted prior in ultrapure water at pH 7.0.

#### 4.3.3. Morphology

The samples were analyzed by means of a transmission electron microscopy (TEM) (Thermo Scientific Talos F200C) operating at 80 KV. For TEM analysis, the samples were placed on copper grids XP-UC300 (Shanghai X-Pivot Optoelectronic Technology Co., Ltd., Shanghai, China). Scanning Electron Microscopy (SEM) was used to determine the structure of the lyophilized FN–SLN. FN–SLN was dispersed in water and spread on a double-sided conducting adhesive tape, then was pasted on a metallic stub. After drying, it was coated (100 mm) with gold in a sputter coating unit for 5 nm using an accelerated voltage of 15 KeV.

### 4.4. Lyophilizated FN-SLN Evaluation

#### 4.4.1. Cryoprotectant Selection

To facilitate freeze-drying and obtain a free-flowing powder with minimal increase in particle size (PS), different cryoprotectants (glucose, mannitol, and trehalose) were evaluated at different concentrations. 10% and 20% (*w*/*w*) of cytoprotectant were dissolved in water at room temperature with magnetic stirring. The SLN dispersion was then mixed with the cytoprotectant solution in a 1:1 ratio. The mixed solution was subjected to deep freezing at a temperature of −40 °C for a period of 36 h. The frozen nanoparticle was subjected to lyophilization at room temperature and a 0.001 mbar vacuum using a Labconco freezone 6L-84 lyophilizer (Labconco corporation, Kansas City, MI, USA).

#### 4.4.2. FN–SLN Encapsulation Efficiency and Loading Capacity Assessment

Weight 5 mg fucoxanthin SLN powder and add 3 mL hexane and vortexed for 1 min, followed by centrifuging at 10,000 rpm at 4 °C for 10 min. The upper hexane layer was collected, evaporated to dryness, re-dissolved in methanol: water (90:10, *v*/*v*), and analyzed by HPLC. EE was calculated using the equations below:

(1) $$EE\left(\%\right)=\left(1-\frac{Free\;FN\;on\;the\;surface\;of\;SLNs}{Total\;FN\;in\;freeze-dried\;SLNs\;powder}\right)\times 100\%$$

Weight 3 mg FN-SLN freeze-dried powder and add 200 µL of ultrapure H_2_O, vortexed for 2 min to dissolve the cryoprotectant-wrapped layer, after that, 2800 µL of methanol was added to extract the FN in the SLNs. The solution was then filtered through a 0.22 µm membrane for HPLC analysis, calculated with the following equation:

(2) $$LC\left(\%\right)=\frac{Total\;amount\;of\;FN-free\;FN\;on\;the\;surface\;of\;SLNs}{\left(weight\;of\;freeze-dried\;FN-SLN\;powder\right)\times lipid\;percentage}\times 100\%\;$$

#### 4.4.3. In Vitro Release Study

A 3 mL dispersion of solid lipid nanoparticles (SLNs) and fucoxanthin (FN) crystal at a concentration of 1 mg/mL/FN was placed in separate dialysis cassettes (Slide-A-Lyzer, Thermo Fisher Scientific, USA) with a molecular weight cutoff (MWCO) of 20 KDa. Each cassette was then immersed in 120 mL of release medium, consisting of 60% ethanol and 40% SGF, and maintained at 37 °C with a stirring speed of 120 rpm for the first 2 h. An appropriate amount of ethanol is necessary to be added to improve the release of FN in a water-based environment [28]. Two hours later, SGF was replaced with SIF. Samples of 10 µL were periodically withdrawn from the internal compartment of the dialysis cassette, using methanol for extraction and dilution to determine the concentration of fucoxanthin. The remaining FN% was calculated with the following equation:

(3) $$Releasing\;FX\left(\%\right)=(1-\frac{M_{t}}{M_{0}})\times 100\%$$

where: *M*_0_ is the initial amount of FN, and *M_t_* is the amount of FN calculated at a given incubation time.

#### 4.4.4. In Vitro Dissolution Test

In this study, 7.8 mg of FN–SLNs and FN crystals were encapsulated in separate enteric-coated capsules. The capsules were placed in a glass bottle containing 2 mL of SGF and maintained at 37 °C, stirring at 120 rpm for the first 2 h. During this period, 100 µL samples were collected at predetermined intervals to assess the dissolution profiles of both formulations. Each time a sample was taken, 100 µL of the corresponding fluid was added to maintain the total volume. After 2 h, the capsules remained intact, demonstrating effective protection against the acidic environment. Following this, the SGF was replaced with 2 mL of SIF, and sampling continued at specified intervals. After each sampling, the collected solutions were centrifuged for 2 min (5000 rpm, 4 °C) to separate any undissolved particles. The supernatants were then extracted using methanol, allowing for the quantification of FN using high-performance liquid chromatography (HPLC).

#### 4.4.5. In Vitro Cytotoxicity Test

The cytotoxicity of FN–SLNs was evaluated on the keratino cytes (HaCaT) cell line using the CCK-8 assay. At 24 h before the cytotoxicity test, the cells were detached from the growth flasks by trypsinization at 37 °C for 2 min. Then, 5 × 10^4^ cells were seeded into each well of a 96-well Nunc plate with a concentration of 100 μL of medium per well and cultured in a 37 °C, 5% CO_2_ incubator for about 24 h until a monolayer of cells was formed. Subsequently, the medium was removed, and the cells were washed with PBS. The dilutions of the test compounds prepared directly in the medium were added to each well of the 96-well plate with a volume of 100 μL. The tested concentrations were 1, 20, 50, 100, 200, and 500 μg/mL. After co-culturing with the test compounds for 24 h, the medium was removed and 100 μL of medium containing 10% CCK-8 was added, followed by further incubation at 37 °C for 3 h. The absorbance was recorded at a wavelength of 450 nm by the microplate plate reader (Varioskan LUX, Thermo Fisher Scientific Inc., MA, USA). Six replicates were performed for each FN–SLN concentration. A blank sample was used to subtract the background absorbance. Finally, the viability was calculated as the percentage using the following formula:

(4) $$Cell\;viability\;\left(\%\right)=\frac{OD\left(treatment\right)-OD\left(blank\right)}{OD\left(control\right)-OD(blank)}\times 100\%$$

#### 4.4.6. In Vivo Pharmacokinetics Test

- Animal prepara

Male rats (SD, 300 ± 20 g) were obtained from Zhuhai BesTest Bio-Tech Co., Ltd. All animal treatments were approved by the Institutional Animal Care and Use Committee (refer to: NIH Publications No. 8023, revised 1978) and were carried out according to the Laboratory Animal Management Regulations of the People’s Republic of China (edition 2017), as well as complying with institutional guidelines.

The rats (aged 6–8 weeks) were housed in standard laboratory conditions with a controlled temperature (23 ± 2 °C) and a 12-h light-dark cycle. They were provided with standard rat chow and water and libitum throughout the study. The rats were acclimatized to the laboratory environment for 7 days prior to the start of the experiment to minimize stress-induced effects.

The in vivo bioavailability of FN-SLNs was assessed through a pharmacokinetic study conducted in SD rats with a dosage of 10 mg/kg (based on FN). Twelve clean-grade male SD rats were randomly divided into two groups and fasted for 12 h. Each group received intragastric administration of FN–SLNs and FN at a dosage of 10 mg/kg. Prior to blood collection, the rats were anesthetized with isoflurane. Blood samples were collected via orbital blood collection at the following points: 30 min, 1, 2, 4, 6, 8, 10, 12, 24 h post-administration. After collecting, the blood samples were centrifuged down to obtain plasma by separation and then stored frozen at −80 °C until further analysis.

- Sample Analysis

60 μL of rat plasma was accurately transferred into a 1.5 mL brown Eppendorf tube. Then, 540 µL of precooled methanol (containing the internal standard at 2 ng/mL) was added and vortexed for 2 min for subsequent extraction. Centrifugation was conducted at 12,000 rpm for 10 min with 4 °C. The supernatant was carefully collected (450 µL) and filtered with 0.22 μm filter membrane for analysis using LC-MS/MS.

The plasma drug concentration data were fitted using a non-compartmental model, and pharmacokinetic parameters were calculated via Phoenix WinNonlin 8.1 (Certara Corporation, Princeton, NJ, USA). The PK graph was plotted using a GraphPad Prism (10.3.1). By using FN crystal suspension as the reference, the relative bioavailability (Frel) of FN–SLNs was determined using the following formula:

(5) $$\;F\left(relative\right)\%=\frac{AUC\;\left(FN-SLN\right)}{AUC\left(FN\;crystal\right)}\times 100\%$$

## 5. Conclusions

The FN–SLN formulation developed in this study has demonstrated outstanding achievements in multiple aspects. Through meticulous formulation optimization, it achieved excellent stability, favorable in vitro release characteristics, and showed no cytotoxicity in vitro while presenting remarkable in vivo pharmacokinetics. These findings strongly indicate that the FN–SLN formulation not only effectively enhances the bioavailability of fucoxanthin but also represents a highly promising and innovative delivery system. It serves as a robust foundation for the extensive application of fucoxanthin in both functional foods and pharmaceuticals and simultaneously paves the way for further exploration and expansion of solid lipid nanoparticle applications.

Although this study has achieved significant progress, there are still areas for improvement and further exploration. The current FN–SLN formulation holds the potential for continuous optimization, with the objective of attaining enhanced drug-loading capacity and ensuring more prolonged term stability. The in vivo evaluation of enteric-coated FN–SLNs will present an opportunity for deeper understanding and optimization. Additionally, expanding the cytotoxicity assessment to a broader range of cell lines and tissues will enhance our understanding of its safety profile, providing more reliable data and support for its clinical translation. Future studies could focus on exploring the therapeutic potential of this formulation in relevant biological models. By doing so, we can further unlock the hidden potential of FN–SLN and move closer to realizing its full clinical implications and benefits, ultimately making a more significant contribution to the field of fucoxanthin-based biomedical applications.

## Figures and Tables

**Table 1:** Factors and levels of L9 (3^4^) orthogonal design for FN–SLNs formulation.

Factors	Level 1	Level 2	Level 3
A: Lipid (HPO: Coconut oil)	0%	50%	100%
B: Surfactant	Tween 80	soy lecithin	glyceryl monostearate
C: Surfactant Concentration	1%	2.5%	5%
D: HPH cycles	3	4	5

**Table 2:** The optimization of FN–SLN formulation parameters by the orthogonal experiment. (*n* = 3, mean ± SD).

No.	Factor A	Factor B	Factor C	Factor D	Particle Size (nm)	PDI	Zeta Potential (mV)	EE%
1	1	1	1	1	157.10 ± 3.19	0.183 ± 0.011	−32.50	96.01%
2	1	2	3	2	156.37 ± 6.80	0.191 ± 0.032	−58.66	97.45%
3	1	3	2	3	360.05 ± 11.31	0.178 ± 0.012	−31.72	96.21%
4	2	1	3	3	148.52 ± 2.42	0.251 ± 0.190	−24.16	97.29%
5	2	2	2	1	246.19 ± 7.65	0.226 ± 0.167	−49.21	95.45%
6	2	3	1	2	326.99 ± 10.01	0.305 ± 0.020	−35.01	94.76%
7	3	1	2	2	256.05 ± 3.17	0.262 ± 0.031	−31.92	90.72%
8	3	2	1	3	235.86 ± 6.80	0.125 ± 0.023	−40.16	89.63%
9	3	3	3	1	1316.59 ± 9.90	0.302 ± 0.020	−33.74	87.78%
K average (1)	224.51 ± 5.41	187.22 ± 4.63	239.98 ± 4.14	573.29 ± 5.10				
K average (2)	240.57 ± 3.13	212.81 ± 5.41	287.43 ± 3.20	246.47 ± 6.71				
K average (3)	602.83 ± 5.60	667.88 ± 4.83	540.49 ± 6.31	248.14 ± 3.11				
Optimum	A1	B1	C1	D2				

**Table 3:** Effects of various cryoprotectants at different concentrations for freeze-drying.

No.	Cryoprotectant	Conc (% *w*/*w* to H_2_O)	Appearance
1	Glucose	10	sticky powder
2	Glucose	20	hygroscopic powder
3	Mannitol	10	sticky powder
4	Mannitol	20	lumpy powder
5	Trehalose	10	hygroscopic powder
6	Trehalose	20	free-flowing powder

**Table 4:** Fucoxanthin SLNs during storage: particle size, PDI, and zeta potential measurement (*n* = 3; mean ± SD).

T = 4 °C	Day 1	Day 10	Day 20	Day 30	Day 90
particle size (nm)	259.51 ± 3.65	262.04 ± 8.90	264.01 ± 4.47	274.14 ± 15.64	303.53 ± 5.18
PDI	0.25 ± 0.007	0.24 ± 0.04	0.21 ± 0.006	0.27 ± 0.13	0.27 ± 0.007
zeta potential (mV)	−33.45 ± 0.48	−39.69 ± 1.47	−32.76 ± 0.33	−35.35 ± 0.90	−36.09 ± 0.48
EE%	97.87 ± 0.14	96.61 ± 0.15	96.64 ± 0.29	95.82 ± 0.93	96.28 ± 0.22

**Table 5:** Pharmacokinetic parameters for FNOH after oral dose of FN and FN–SLNs.

Parameter	Unit	FN-SLN	FN
Cmax	ng/mL	53.63 ± 19.11	2.14 ± 1.04
Tmax	h	3.0 ± 1.15	7.0 ± 1.09
AUC_0-last_	ng·h/mL	536.19 ± 164.77	19.69 ± 12.51
AUC_0-inf_	ng·h/mL	595.37 ± 207.17	43.99 ± 12.53
T1/2	h	5.29 ± 2.90	14.46 ± 3.49
CL	L/h/kg	18.18 ± 6.08	239.183 ± 63.28
Vz	L/kg	123.69 ± 30.27	5203.58 ± 2463.96

(*n* = 6; mean ± SD).

**Table 6:** ESI–MS/MS parameters for fucoxanthin, fucoxanthinol, and hydroxyprogesterone caproate as internal standard.

Analyte	Q1/Da	Q3/Da	Dwell Time/ms	DP/V	CE/V
Fucoxanthin	659.4	581.4, 109.0	100	45	19, 30
Hydroxyprogesterone caproate	429.3	313.4	100	30	20
Fucoxanthinol	617.2	581.4, 109.0	200	20	20,30

## Data Availability

The raw data supporting the conclusions of this article will be made available by the authors on request.

## Supplementary Materials

The following supporting information can be downloaded at: https://www.mdpi.com/article/10.3390/ijms252312825/s1.
