# Supplementary material for: Innovative Approaches to Fucoxanthin Delivery: Characterization and Bioavailability of Solid Lipid Nanoparticles with Eco-Friendly Ingredients and Enteric Coating
Source: Int J Mol Sci. 2024 Nov 28;25(23):12825. doi: 10.3390/ijms252312825 (PMC11641657; doi:10.3390/ijms252312825)

Supplementary

Table S1: Comparison of nanoparticle characteristics before and after lyophilization (n=3)

	pre-lyophilized			Average	SD	post-lyophilized			Average	SD
size (nm)	252.10	250.38	244.47	248.98	4.00	255.50	260.38	262.65	259.51	3.65
PDI	0.26	0.26	0.25	0.25	0.01	0.21	0.22	0.21	0.21	0.01
Zeta potential(mV)	-33.45	-33.93	-32.97	-33.45	0.48	-34.19	-31.37	-32.73	-32.76	1.41
EE%	98.02	98.53	98.35	98.30	0.26	97.87	97.73	98.01	97.87	0.14

Table S2. Cytotoxicity study of various FN - SLN concentrations in HaCaT cell line (n=6)

FN-SLN concentration (ug/ml)	Cell viability (%)
1	99.93±3.53
20	101.58±2.58
50	100.31±7.89
100	100.44±8.56
200	98.49±7.08
500	98.93±6.17

Figure S1: Chromatogram of three analytes in sequence from left to right: Fucoxanthinol, Hydroxyprogesterone caproate and Fucoxanthin

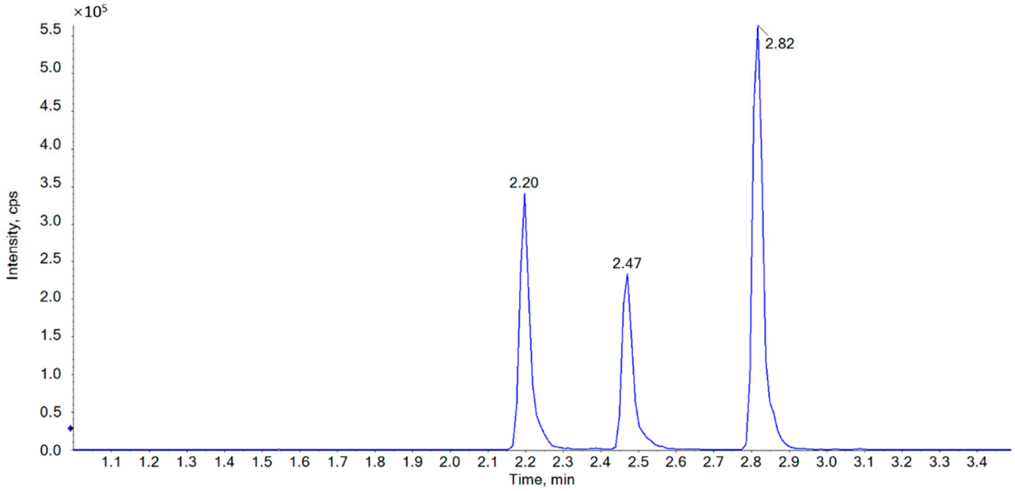

Figure S2: Calibration curve used for FNOH quantitation in rat plasma

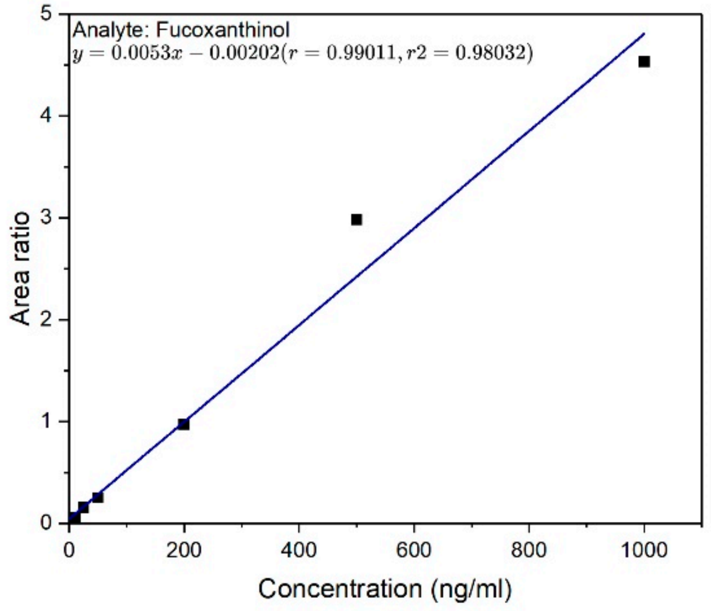

Supplement: Supplementary file 1 [file ijms-25-12825-s001.zip › ijms-3310453-supplementary.pdf]
